# Re-evaluating patient isolation policies for musculoskeletal infections in orthopaedic practice: a scoping review

**DOI:** 10.5194/jbji-10-489-2025

**Published:** 2025-11-28

**Authors:** Laura Bessems, Jolien Onsea, Baixing Chen, Marjan Wouthuyzen-Bakker, Irene K. Sigmund, Tristan Ferry, Richard Kuehl, Martin Clauss, Alex Soriano, Ricardo Sousa, Annette Schuermans, Willem-Jan Metsemakers

**Affiliations:** 1 Department of Development and Regeneration, KU Leuven, Leuven, 3000, Belgium; 2 Department of Trauma Surgery, University Hospitals Leuven, Leuven, 3000, Belgium; 3 Department of Medical Microbiology and Infection Prevention, University Medical Center Groningen, Groningen, the Netherlands; 4 University of Groningen, Groningen, 9713 GZ, the Netherlands; 5 Department of Orthopaedics and Traumatology, Medical University of Vienna, Vienna, 1090, Austria; 6 Infectious and Tropical Diseases Unit, Croix-Rousse Hospital, Hospices Civils de Lyon, Lyon, 69004, France; 7 Division of Infectious Diseases & Hospital Epidemiology, University Hospital Basel, Basel, 4031, Switzerland; 8 Department of Orthopaedics and Trauma Surgery, Center for Musculoskeletal Infections (ZMSI), University Hospital Basel, Basel, 4031, Switzerland; 9 Hospital Clinic of Barcelona, Barcelona, 08036, Spain; 10 IDIBAPS, Barcelona, 08036, Spain; 11 CIBERNIF, CIBER in Infectious Diseases, ISCIII, Madrid, 28029, Spain; 12 Orthopaedic Department, Porto Bone Infection Group (GRIP), ULS Santo António, Porto, 4099-001, Portugal; 13 Department of Infection Control and Epidemiology, University Hospitals Leuven, Leuven, 3000, Belgium; 14 Department of Public Health and Primary Care, KU Leuven – University of Leuven, Leuven, 3000, Belgium

## Abstract

**Introduction**: Historically, isolating patients diagnosed with musculoskeletal infections (MSIs) from the general orthopaedic population has been regarded a fundamental aspect of effective infection control. However, this remains controversial. Evolving perspectives on infection prevention, resource constraints, and staffing shortages necessitate a reassessment of current practices. This scoping review examines existing isolation policies for MSIs in orthopaedic practice and provides expert recommendations for hospital policymakers. **Materials and methods**: A systematic search of seven databases identified 23 320 articles. After deduplication and screening of 10 621 abstracts, 119 full texts were reviewed and 14 studies met the inclusion criteria. A total of 9 studies involved surgical wards, 5 examined general hospital wards, and 2 addressed orthopaedic patients. **Results**: Evidence indicates that individual isolation measures can reduce methicillin-resistant *Staphylococcus aureus* infections, whereas additional contact precautions or isolation showed no reduction of transmission risk for extended-spectrum beta-lactamase-producing *Enterobacterales* in endemic settings. For vancomycin-resistant *Enterococcus* (VRE), one study found a reduction in infections after implementing individual isolation, while another study reported no impact. No evidence supports separating patients with non-resistant MSIs from elective orthopaedic patients. Similarly, no data support the routine use of dedicated septic wards in orthopaedic practice. **Conclusions**: Effective infection control relies on hospital-wide strategies, provided that appropriate preventive measures and a high level of compliance with standard precautions are in place. Isolation practices should be selectively tailored to local epidemiology to balance infection prevention with optimal resource utilization. Managing MSIs in specialized centres, instead of dedicated septic wards, may deliver more effective care and adherence to standard precautions.

## 1 Introduction

The isolation of patients with infectious diseases has been a long-established practice in healthcare. Within orthopaedics, this historically entailed the utilization of dedicated septic wards and specialized hospitals to ensure that infected patients were segregated from those undergoing elective procedures, thus reducing the risk of cross-contamination (Kempf et al., 1985). Such strict protocols were deemed essential because infections in orthopaedic surgery present a significant challenge due to the presence of lifelong implants, which can act as a persistent predisposing factor of infection. These measures were further driven by limited treatment options and the significant threat of infectious disease outbreaks. However, the introduction of antibiotics and the later development of the principles of standard precautions fundamentally changed infection prevention strategies in modern healthcare, shifting the focus by the late 20th century away from structural patient separation (e.g. septic wards) toward the universal application of standard precautions. This shift marked a pivotal change in hospital infection control, integrating prevention more deeply into routine care rather than relying solely on physical isolation (Prevention CDC, 2007; Haynes and Khardori, 2013).

Today, the practice of isolating patients with confirmed infections is conventionally used to prevent the transmission of pathogens within healthcare environments. Measures such as single-patient rooms, hand hygiene, environmental hygiene, and transmission-based precautions aim to minimize transmission, especially in postoperative and vulnerable populations such as immunocompromised patients (Allegranzi et al., 2016; Cooper et al., 2004; Prevention CDC, 2007). However, in modern healthcare, this is usually reserved for patients with highly transmissible or multidrug-resistant organisms (MDROs), such as methicillin-resistant *Staphylococcus aureus* (MRSA) or carbapenamase-producing Gram-negative bacilli. These organisms are linked to both colonization and infection, and they are prioritized for isolation due to their potential for transmission, restricted therapeutic options, and the significant risks of cross-contamination, especially in high-risk surgical or orthopaedic patients. For most other infecting agents, the relevance and effectiveness of certain strategies, like patient isolation, are being questioned.

This is particularly relevant in the aftermath of the COVID-19 pandemic, which exposed resource limitations and staff shortages that further complicated infection management. As healthcare systems continue to evolve, a re-evaluation of isolation protocols in routine orthopaedic practice is necessary to determine whether traditional isolation strategies remain effective or if alternative, more rigorous approaches should be prioritized.

Therefore, this scoping review aims to evaluate the necessity of classic isolation policies for patients with musculoskeletal infections (MSIs) in orthopaedic practice. Several research questions have been formulated to examine this topic in detail. The answers to these questions were based on the best available evidence and expert opinion.

## 2 Research questions

1. *Ward-level isolation strategies*
  What evidence supports the implementation of septic wards for isolating patients with MSIs, aimed at preventing cross-contamination in orthopaedic practice?
2. *Patient isolation policies*
  a. What evidence supports isolating patients with MSIs caused by MDROs (e.g. MRSA) in single-patient rooms?
  b. What evidence supports isolating patients with MSIs caused by non-MDROs (e.g. methicillin-sensitive *Staphylococcus aureus* (MSSA)) in single-patient rooms?
  c. What is the evidence of cohorting patients with MSIs caused by non-MDROs (e.g. MSSA) from patients undergoing elective orthopaedic procedures (e.g. arthroplasty) on a ward level?
3. *General infection control measures*
  What evidence supports the exclusive use of standard precautions as a single measure compared to transmission-based precautions, including isolation, for preventing cross-contamination in orthopaedic practice?

## 3 Methods

This scoping review was conducted in accordance with the PRISMA-ScR guidelines, with the objective of determining whether patients with MSI require transmission-based precautions, such as isolation, in hospital wards. We structured our inclusion and exclusion criteria based on the Joanna Briggs Institute (JBI) Population, Concept, Context (PCC) framework, which is summarized in Table 1.

**Table 1:** JBI PCC framework for inclusion and exclusion criteria.

	Description
Population	Infected or colonized patients admitted to the surgical ward
Concept	Isolation policies
Context	Hospital ward setting

Both colonized and infected MSI patients were included to mirror common study practices. Furthermore, we distinguished between cohorting (*all MSI patients grouped on a dedicated unit*) and single-room isolation with transmission-based precautions. During data extraction, we recorded the precise terminology each study employed and catalogued all described infection prevention and control measures.

### Search strategy and selection criteria

The search strategy was designed in collaboration with biomedical reference librarians. The initially developed PubMed strategy, based on MeSH terms and keywords related to “patient isolation” and “postoperative nosocomial infections”, was adapted for Embase, Scopus, Web of Science, Cochrane (CENTRAL and CDSR), CINAHL, and ClinicalTrials.gov. The search was completed on 8 May 2025. Non-English articles, preclinical studies, and reviews were excluded. Records were deduplicated in Endnote 21, Clarivate Analytics, US, and screened in Rayyan by two independent reviewers (LB, BC), with disagreements resolved by a third (WJM) (Ouzzani et al., 2016). The included articles were then subjected to data extraction and analysis in accordance with the objectives of the review.

An Open Science Framework (OSF) protocol was published for this scoping review.

## 4 Results

**Figure 1:**
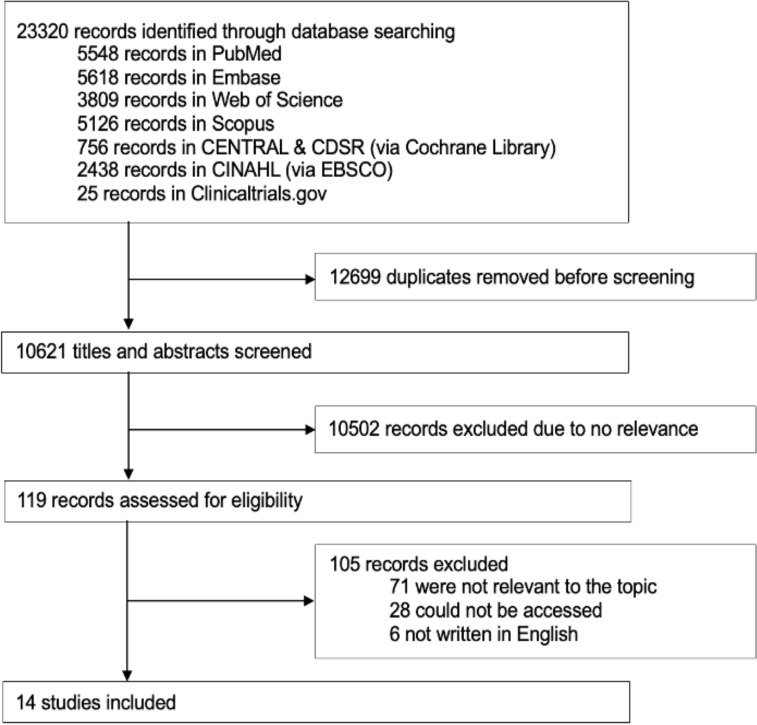
Study selection.

The database search resulted in a total of 23 320 articles. After deduplication, 10 621 abstracts were screened using Rayyan according to predefined eligibility criteria. After abstract screening, 119 studies were selected for full-text review. Upon further assessment, 14 articles met the inclusion criteria for this review (PRISMA flow diagram Fig. 1).

### 4.1 Surgical specialties represented in the literature

Of the 14 included studies, 9 focused on patient isolation practices within surgical wards, mainly cardiothoracic surgery (5 studies) (Blane et al., 2023; Carrier et al., 2002; Mastoraki et al., 2008; Schelenz et al., 2005; Yavuz et al., 2013); only 2 studies specifically investigated orthopaedic patients (Kawamura et al., 2016; Talon et al., 2003). Other studies included mixed surgical populations or combined medical–surgical units, limiting the specificity of conclusions for individual departments (Tables 2–3).

**Table 2:** Studies assessing the impact of patient isolation in the surgical ward.

Author	Study design	Population	Intervention	Result
Blane et al. (2023)	Prospective genomic surveillance study	VRE colonized/infected patients admitted at the CCU or cardiothoracic surgical ward	– Move from a hospital built in 1918 to a new hospital with close to 100 % single-patient rooms – Environmental screening – VRE screening – Clinical infection samples	– Significant reduction in positive environmental screening swaps pre-move (28.9 %) vs. post-move (1.0 %) – The rate of VRE carriage/infection almost halved post-move
Carrier et al. (2002)	Retrospective case-control study	MRSA-infected patients post-cardiac surgery	– Nasal screening of all surgical patients – Preventive isolation of all carriers in a private room – Mupirocin ointment applied to the nares of carriers – Vancomycin antibiotic prophylaxis for cardiac surgery of infected patients	– The incidence of MRSA mediastinitis decreased significantly – The rate of non-MRSA mediastinal infection did not vary significantly during the study period
Curran et al. (2006)	Prospective cohort study	MRSA-colonized/MRSA-infected patients in the vascular surgery ward	– Introduction of a cohort unit was prompted by a persistent rise in MRSA cases under standard infection control measures – All new admissions were placed in an isolation room and screened – Patients with a positive screening for MRSA were transferred to the cohort unit	– Significant reduction in nosocomial MRSA isolates after the introduction of a cohort unit – After the cohort unit was discontinued, the number of new MRSA-colonized/MRSA-infected patients did not return to pre-cohort levels
Kawamura et al. (2016)	Retrospective before–after study	Orthopaedic MRSA SSI patients	– Preoperative nasal screening in all patients before or at admission to the orthopaedic ward – Mupirocin treatment in MRSA-positive patients – Single-room isolation or cohorting only for confirmed MRSA infection patients (period A) vs. single-room isolation or cohorting for all MRSA colonized/infected patients (period B) – New antibiotic prophylaxis protocol implemented in period B	– The MRSA SSI rate during period B was significantly lower than that during period A – The infection rate in MRSA carriers did not differ between the two periods – Cefazolin antimicrobial use density is negatively correlated with the rate of MRSA SSIs – Prolonged ( > 48 h) antimicrobial prophylaxis is a risk factor for MRSA SSIs
Maechler et al. (2020)	Cluster- randomized crossover trial	ESBL-E-colonized/ESBL-E-infected patients admitted at the adult medical and surgical wards	– ESBL-E carriage screening within 3 d of admission, once a week thereafter and on discharge – Periods of standard precautions vs. periods of contact precautions and isolation in addition to standard precautions for patients (previously) colonized/infected with an ESBL-E	– Contact precautions and isolation showed no benefit when added to standard precautions – Higher baseline ESBL-E prevalence on initial screening was positively associated with ESBL-E acquisition (i.e. more existing carriers led to a higher risk of new cases) – Greater screening coverage was negatively associated with ESBL-E acquisition (i.e. more extensive screening led to a lower risk of new cases)

**Table 2:** Continued.

Author	Study design	Population	Intervention	Result
Mastoraki et al. (2008)	Prospective observational cohort study	MRSA in patients after cardiovascular surgical procedures	– Nasal screening for all patients admitted from other hospitals or being at risk for developing infectious complications – Patients at risk remained isolated until proved MRSA-negative – All newly employed healthcare workers were screened for MRSA – Preoperative de-colonization with nasal mupirocin after MRSA identification – Barrier precautions used in each MRSA-positive patient	– Successful control of MRSA spread
Schelenz et al. (2005)	Retrospective observational study	MRSA infections in cardiothoracic patients	– Introduction of an enhanced infection control programme – Improved hand hygiene – Weekly MRSA screening and decontamination – Specialized team nursing for colonized patients – MRSA patients isolated in single rooms	– Significant decrease in the proportion of patients acquiring MRSA on the ward and in the rate of bloodstream MRSA infections after the introduction of the enhanced programme – Sternal and wound infections both halved but did not reach statistical significance – Not clear which of the interventions are critical for success
Talon et al. (2003)	Retrospective cohort study	Orthopaedic patients with clinical specimens positive for MRSA	– Introduction of a surgical dedicated cohort facility	– Individuals hospitalized in the dedicated cohort facility have a higher risk of acquiring MRSA due to higher colonization pressure – The risk of acquiring MRSA in the entire hospital, outside of this dedicated cohort facility, is reduced when this type of unit is available
Yavuz et al. (2013)	Prospective surveillance	Patients with sternal SSI after open heart surgery	– Range of 12 infection prevention interventions implemented during a period of 8 years, including isolation or cohorting of patients colonized/infected by MRSA, preoperative nasal screening for MRSA, and decolonization of MRSA-positive patients with mupirocin and vancomycin prophylaxis	– No substantial decrease in the total rate of sternal SSIs – Significant fall in the rates of MRSA and MSSA SSIs – As the 12 interventions were implemented simultaneously, it is impossible to determine the precise impact of individual interventions

VRE: vancomycin-resistant *Enterococcus faecium*; CCU: critical care unit; MRSA: methicillin-resistant *Staphylococcus aureus*; SSI: surgical site infection; ESBL-E: extended-spectrum beta-lactamase-producing *Enterobacterales*; MSSA: methicillin-sensitive *Staphylococcus aureus*.

**Table 3:** Studies assessing the impact of patient isolation in patients with (not exclusively postoperative) infections.

Author	Study design	Population	Intervention	Results
Arruda et al. (2019)	Observational, interrupted time series study	All hospitalized patients who acquired MDR bacteria	– In the first phase, patients who acquired MDR bacteria were isolated without physical transfer from the original hospital unit – In the second phase, patients were transferred to a specific isolation unit	– No reduce of overall risk to acquire MDR bacteria in the second phase – The incidence of some specific microorganisms was higher in the second phase
Chang et al. (2022)		All hospitalized patients colonized or infected with VRE	– In the first phase, isolating patients infected or colonized with VRE in private rooms was maintained (private isolation era) – In the second phase, patients infected or colonized with VRE were isolated in a cohort (cohort isolation era) – In the third stage, no VRE isolation measures were implemented (no isolation era)	– With the maintenance of hand hygiene compliance, the incidence of healthcare-associated VRE bacteraemia did not increase significantly even when the VRE isolation policy was relaxed step by step
Hartstein et al. (1995)	Prospective surveillance study	Hospitalized patients with MRSA colonization/infection	– Barrier isolation of affected and unaffected patients in and admitted to the SICU – Nasal screening of staff in contact with outbreak cases	– Annual frequency of new nosocomial MRSA cases decreased, and only one outbreak caused by type-related isolates occurred
Shanson et al. (1985)	Outbreak study	Hospitalized patients with MRSA colonization/infection	– Strict isolation of colonized/infected patients as well as (negative) contact patients – Increasing the level of staffing on the separate isolation unit	– Control of the outbreak achieved
Tschudin-Sutter et al. (2012)	Observational cohort study	All hospitalized patients colonized or infected with an ESBL-producing pathogen	– Active screening for ESBL carriage in patients hospitalized in the same room as colonized/infected patients – All patients colonized/infected with an ESBL-producing pathogen were assigned to contact precautions exclusively in single rooms	– With use of standard precautions, 2 transmissions (of 133 contact patients) were identified, both resulting in colonization

MDR: multidrug-resistant; SICU: surgical intensive care unit; MRSA: methicillin-resistant *Staphylococcus aureus*; ESBL: extended-spectrum beta-lactamase.

### 4.2 Pathogens examined

The majority of studies focused on MDROs, mainly MRSA (nine studies) (Carrier et al., 2002; Curran et al., 2006; Hartstein et al., 1995; Kawamura et al., 2016; Mastoraki et al., 2008; Schelenz et al., 2005; Shanson et al., 1985; Talon et al., 2003; Yavuz et al., 2013), followed by extended-spectrum beta-lactamase (ESBL)-producing *Enterobacterales* (two studies) (Maechler et al., 2020; Tschudin-Sutter et al., 2012) and vancomycin-resistant *Enterococcus* (VRE) (two studies) (Blane et al., 2023; Chang et al., 2023). Notably, no studies focused on infections caused by non-resistant or common community-acquired pathogens only. One study, by Yavuz et al., considered both MRSA and methicillin-sensitive *S. aureus* (MSSA) in its infection control measures (Yavuz et al., 2013).

### 4.3 Ward-level isolation strategies

Limited data were available on ward-level isolation measures such as dedicated septic units. Seven studies implemented cohort units for MDRO-colonized or MDRO-infected patients (Arruda et al., 2019; Curran et al., 2006; Mastoraki et al., 2008; Shanson et al., 1985; Talon et al., 2003; Tschudin-Sutter et al., 2012; Yavuz et al., 2013). Curran et al. (2006) introduced a cohort unit for MRSA-positive patients, leading to a significant reduction in nosocomial MRSA incidence ( p=0.0005 ). However, after discontinuing the cohort unit, MRSA rates remained lower than before implementation, suggesting a lasting benefit of other concurrent measures. Talon et al. (2003) reported that while a dedicated surgical cohort for all MSI patients reduced MRSA acquisition hospital-wide, it increased colonization pressure with MRSA within the cohort, potentially increasing the risk of MRSA acquisition for those patients. No studies assessed the value of septic units for patients with infections caused by non-MDROs (e.g. MSSA).

### 4.4 Individual isolation strategies

Single-room isolation, particularly when combined with targeted infection control interventions (e.g. preoperative screening policy, MRSA decolonization with mupirocin), was associated with reduced MRSA transmission and infection incidence in both general hospital wards and orthopaedic wards. Kawamura et al. (2016) found that expanding isolation from MRSA-infected patients to both colonized and infected patients significantly reduced the rate of MRSA MSIs from 2.17 % ( 29/1333 ) before the intervention to 0.97 % ( 19/1966 ) after the intervention ( p = 0.003) (Kawamura et al., 2016). Schelenz et al. (2005) described a multimodal intervention (e.g. single-room isolation, hand hygiene, weekly MRSA screening) that significantly decreased MRSA acquisition ( 38/1036 to 14/921 ; p=0.003 ) and bloodstream infections ( 12/1075 to 2/956 ; p=0.014 ) (Schelenz et al., 2005). Similarly, Blane et al. (2023) observed that transitioning to a predominantly single-patient room facility nearly halved VRE carriage and infection rates (10.9 to 6.2 cases per 10 000 bed days; p=0.005 ). However, contrasting evidence exists: Chang et al. (2023) observed that relaxation of single-room policy, provided that other infection control measures (e.g. hand hygiene, contact precautions by wearing gloves and gowns, environmental disinfection, and antibiotic stewardship) were maintained, did not lead to an increase in VRE infections (incidence rate ratio 0.99, 95 % CI 0.77–1.26, p=0.903 , when comparing the cohort-isolation era to the no-isolation era). In the study by Maechler et al. (2020), contact isolation involved placing ESBL-*Enterobacterales* carriers in either single-bed rooms or cohorted multibed rooms, with staff required to wear gowns and gloves for all patient interactions. In contrast, standard precautions emphasized strict hand hygiene and the use of protective equipment only when necessary (e.g. during contact with bodily fluids). Their cluster-randomized crossover trial, which included 11 368 patients, found no significant difference in ward-acquired ESBL-*Enterobacterales* cases between the two approaches: 368 cases occurred during contact isolation periods compared to 369 under standard precautions (incidence densities 6.0 vs. 6.1 per 1000 patient days; p=0.97 ). Likewise, Tschudin-Sutter et al. (2012) reported minimal transmission, with only two cases among 133 contacts when standard precautions were combined with single-patient room isolation (Tschudin-Sutter et al., 2012).

### 4.5 General infection control measures

Across surgical and general hospital settings, standard precautions (e.g. staff engagement, preoperative screening) were consistently linked to decreased rates of healthcare-associated infections. Schelenz et al. (2005) and Yavuz et al. (2013) emphasized that combined hygiene interventions, including hand hygiene, MRSA screening, and staff training, significantly reduced MRSA *and* MSSA infections in patients undergoing cardiothoracic surgery. In the study by Yavuz et al. (2013), infection control measures (e.g. preoperative nasal screening, decolonization of MRSA with mupirocin and vancomycin prophylaxis, increased hand hygiene education, and the use of single-patient rooms or cohorting of patients infected or colonized by MRSA) reduced the rate of sternal surgical site infections from 3.63 % to 1.65 % ( p<0.0001 ). However, it remained unclear which specific interventions contributed most to these reductions, as they were often implemented simultaneously. Several studies reported that improved hand hygiene and consistent education of staff compliance had lasting benefits, even without dedicated isolation units (Hartstein et al., 1995; Schelenz et al., 2005; Chang et al., 2023; Maechler et al., 2020; Yavuz et al., 2013).

## 5 Discussion

This review aimed at evaluating the need for patient single-patient room strategies and standard infection control measures in relation to MSIs in routine orthopaedic practice. Most included studies focused on cardiothoracic and vascular surgery; only two investigated orthopaedic patients (Kawamura et al., 2016; Talon et al., 2003), so the recommendations presented below partly rely on extrapolated evidence and expert opinion.

### 5.1 Ward-level isolation strategies

The first research question investigated whether dedicated septic wards are necessary to isolate patients with MSIs and prevent cross-contamination. Our review found no evidence supporting ward-level isolation measures. The commonly held expert opinion is that such ward-level cohorting is not necessary, provided that there is a high level of compliance with standard precautions. Moreover, increased colonization pressure within cohort isolation units has been described in the case of MRSA, potentially increasing infection risk (Talon et al., 2003). Grouping infected patients together increases cross-transmission risk, especially when standard precautions are inconsistent. For example, a patient infected with MRSA may acquire VRE from a neighbouring patient via healthcare worker contact. This means that while ward-level isolation may reduce the risk of transmission to uninfected patients elsewhere in the hospital, it may simultaneously increase the risk for patients already cohorted together. Ideally, cohorting should be pathogen-specific, but even then, molecular differences between strains may limit its effectiveness, and patients who have cleared their original strain may remain vulnerable to re-colonization from other strains within the cohort. Importantly, none of the reviewed studies examined dedicated isolation strategies for infections caused by non-MDROs only. As a result, there is no clear evidence that septic wards provide added value over comprehensive, hospital-wide infection control strategies in routine orthopaedic practice. Given the high stakes of postoperative infectious complications in orthopaedic care, particularly in procedures like joint replacements, any isolation approach must be carefully balanced against its potential to increase colonization pressure.

### 5.2 Individual isolation strategies

The second research question was subdivided into three components, examining the evidence for isolating patients with MSIs caused by MDROs versus non-MDROs in orthopaedic practice. Additionally, it included an inquiry regarding the separation of infected patients from those undergoing elective orthopaedic procedures, a topic that remains contentious within the orthopaedic community. In routine clinical practice, this implies that certain orthopaedic surgeons performing elective surgeries (e.g. joint arthroplasties) may request that their patients do not share rooms with individuals suffering from any type of MSI.

#### 5.2.1 Hospital policy for MDROs and non-MDROs

The first component addressed the evidence for isolating patients with MSIs caused by MDROs in orthopaedic practice. This is a relevant question, as these pathogens, especially MRSA, are linked to extended hospital stays, higher rates of persistent infection and reoperations, and lower treatment success rates, resulting in worse outcomes for orthopaedic patients (Tande and Patel, 2014; Metsemakers et al., 2018). In support of these observations, a comprehensive systematic review by Cooper et al. (2003) provided epidemiological and economic modelling that reinforces the efficacy of targeted isolation measures in reducing MRSA transmission.

In our review, most of the included studies focused specifically on MDRO-related interventions, and the evidence supports a differentiated approach based on the pathogen involved. For instance, Schelenz et al. (2005) reported that a multimodal intervention, combining single-room isolation, enhanced hand hygiene, and weekly MRSA screening, resulted in marked reductions in MRSA acquisition and bloodstream infections. This study emphasized the effectiveness of targeted isolation measures for MDROs. Similarly, Blane et al. (2023) observed that implementing predominantly single-patient rooms led to a substantial reduction in environmental contamination and carriage of VRE. These results highlight the critical role of environmental hygiene, especially regarding pathogens like VRE with considerable environmental persistence. Consequently, thorough disinfection of patient rooms following the discharge of colonized or infected individuals is vital to ensure effective infection prevention measures.

In contrast, findings regarding ESBL-producing bacteria were less consistent. Maechler et al. (2020) and Tschudin-Sutter et al. (2012) indicated that, in settings with high compliance to standard precautions, additional isolation measures may not provide added benefit. It has to be stated that both studies did not focus on surgical patients. These results suggest that escalation of isolation policies may not be warranted for all MDROs and should be tailored to pathogen transmissibility, the resistance profile, and the potential dissemination of the resistance mechanism.

With respect to the second part of this question and as mentioned above, none of the reviewed studies examined dedicated isolation strategies for infections caused by non-MDROs, making it difficult to develop recommendations. However, according to expert opinion, there is no evidence that patient isolation (provided that standard precautions are adequately followed) reduces the spread of infection, even for highly virulent pathogens such as MSSA.

From a biological and epidemiological perspective, there are also compelling reasons to question the benefit of isolating patients with non-MDRO MSIs. Firstly, in many cases of MSIs, the source of infection is the patient's own microbiota, rather than transmission from healthcare workers, the environment, or other patients (Mangram et al., 1999; Long et al., 2024). However, this assumption holds true only when there is a high level of compliance with standard precautions and when the integrity of the sterile field in the operating room is maintained. Under these conditions, the risk of exogenous transmission is significantly reduced, making endogenous sources the predominant cause of infection. Secondly, the time point of infection is typically peri- or intraoperative, with most surgeries taking place on the same day as hospital admission (Depypere et al., 2020). This timing underscores the importance of strict adherence to hygiene protocols and standard precautions, both in the operating room and on the hospital ward. This narrow window limits, but does not eliminate, the opportunity for hospital-acquired colonization from another patient. In addition, operative factors such as surgical technique, procedure duration, and surgeon expertise are critical, highlighting the importance of specialized surgical care to reduce infection risk. Thirdly, about 30 % of the population are persistent nasal *S. aureus* carriers (Kapur et al., 2021). This means that transmission prevention efforts (e.g. isolating one infected patient) may be of limited value, since many other patients (including elective surgery candidates) are already colonized. Taken together, these factors suggest that routine isolation of patients with non-MDRO infections may neither significantly reduce transmission risk nor alter clinical outcomes.

Overall, some studies suggest isolation may reduce care quality and increase adverse events such as falls and pressure ulcers (Stelfox et al., 2003). However, Kang et al. (2022) found no association between the likelihood of adverse events and patient isolation with contact precautions, suggesting that with proper implementation, isolation may not inherently compromise patient safety. Moreover, a quasi-experimental study by Park et al. (2024) on discontinuing single-room isolation for patients with VRE found no significant increase in hospital-acquired VRE bloodstream infections. These findings further support tailoring isolation strategies to the specific clinical context rather than routinely applying resource-intensive measures. Adding to this, although intensive care settings were not included in our review, a prospective two-centre ICU study by Cepeda et al. (2005) compared single-room isolation and cohorting versus standard care for MRSA-positive patients. The study found no significant reduction in MRSA transmission, challenging the assumption that physical isolation always confers additional benefit when standard precautions are strictly followed.

Importantly, isolation of patients infected with MDROs confers benefits that extend beyond the MSI population and apply to all patients within the healthcare environment (Ji and Ye, 2024). Loftus et al. (2018) showed that MRSA carries a significantly higher risk of transmission in the operating room than MSSA, highlighting how targeted isolation protocols for MDRO carriers can reduce perioperative cross-transmission and thus improve safety across both surgical and non-surgical patient cohorts. It should be noted, however, that non-MDRO pathogens, such as MSSA, are also frequently transmitted within healthcare settings. These events often receive less attention, as they generally do not pose therapeutic challenges due to their susceptibility to standard antibiotics. Consequently, their role in nosocomial transmission may be under-recognized in routine surveillance. In contrast, MRSA and other MDROs are more prevalent in hospital environments than in the community, further justifying the implementation of specific isolation precautions to mitigate the risk of healthcare-associated transmission.

#### 5.2.2 Hospital policy with respect to elective surgeries

The third component of this question examined whether patients undergoing elective orthopaedic procedures (e.g. total hip arthroplasty) can be accommodated in the same room as patients with MSIs caused by non-MDROs. The literature did not directly address infection control outcomes in the context of separating these non-MDRO infections. However, in general, guidance from the World Health Organization (WHO) emphasizes the importance of consistent hand hygiene, adherence to standard precautions, and diligent wound care as foundational measures for preventing transmission in healthcare settings, including ward-based surgical site infection prevention measures such as standardized postoperative wound surveillance, timely dressing changes, reinforcing hand hygiene at the bedside, and educating staff on early surgical site infection recognition as outlined in the 2017 CDC update on surgical site infection prevention (Leaper and Edmiston, 2017; Berríos-Torres et al., 2017). This suggests that when such core infection prevention protocols are rigorously applied, the risk of cross-contamination among elective patients in shared environments remains low. It should be noted, however, that teaching hospitals may present an increased risk of cross-transmission due to the high number of individuals involved in patient care, including medical students, residents, and visiting staff. These individuals may have varying levels of experience with infection control practices, and mistakes in following standard precautions can more easily occur in such dynamic clinical environments. Therefore, in facilities with high compliance to the standard precautions, routine physical separation of elective orthopaedic patients from those with treatable, non-resistant infections does not seem necessary. While not evidence-based, separation of infected and elective patients may be regarded as a risk mitigation measure in high-risk units such as orthopaedic wards, provided it does not compromise resource allocation or quality of care.

### 5.3 Integrating general infection control measures

The third and final question evaluated the implementation of general infection control measures (e.g. hand hygiene) to prevent cross-contamination in orthopaedic practice. While organism-specific strategies are essential, a recurring theme across the studies was the importance of comprehensive infection control bundles. Schelenz et al. (2005) and Yavuz et al. (2013) demonstrated that bundled interventions (e.g. rigorous hand hygiene, environmental cleaning, staff training, and targeted screening) markedly reduced the occurrence of nosocomial infections due to cross-contamination in surgical populations. While the individual contribution of patient isolation remains difficult to disentangle from these multifaceted approaches, the evidence suggests that a comprehensive, integrated infection control strategy is essential.

International guidelines reinforce the importance of bundled infection control strategies. Metsemakers et al. (2017) reviewed methods for preventing fracture-related infection and noted the significance of a standardized care package that involves perioperative hand hygiene, timely antibiotic prophylaxis, surgical discipline, and appropriate wound care. These measures are intended to be part of a coordinated approach rather than relying solely on patient isolation. Likewise, the 2022 SHEA/IDSA/APIC update by Glowicz et al. (2023) underscores that hand hygiene remains the most effective strategy for preventing healthcare-associated infections. Furthermore, the cross-border analysis by Cimen et al. (2025) comparing infection prevention guidelines in the northern Dutch–German region emphasizes the value of harmonized protocols, suggesting that coordinated transnational approaches can enhance MDRO management even when national policies vary. In addition to hand hygiene, environmental hygiene plays a critical role in reducing microbial burden on high-touch surfaces and maintaining a safe care environment. Effective cleaning protocols, regular disinfection schedules, and monitoring of cleaning quality are essential to prevent indirect transmission of pathogens in both surgical and non-surgical settings (Dancer, 2009).

The safe reduction of isolation measures, however, requires certain critical conditions to be met and are only appropriate in settings where infection prevention protocols are thoroughly embedded into clinical practice and where healthcare personnel received adequate training and demonstrate high compliance with hygiene standards. Facilities must ensure ongoing education, auditing, and feedback mechanisms to maintain awareness and adherence to infection control principles. In centres where these structural and cultural components are not in place, weakening isolation protocols may lead to unintended risks, including increased transmission.

### 5.4 Post-pandemic healthcare challenges

Given the increasing strain on healthcare systems, exacerbated by post-COVID-19 staff shortages and resource limitations, modern infection control should focus on targeted, evidence-based strategies that balance patient safety with operational efficiency (Armstrong, 2025; Deakin, 2022). High-quality prospective studies are needed to better define the role of isolation policies in preventing postoperative infections. Until then, healthcare providers should invest in knowledge of and compliance with standard precautions to guide case-specific decisions.

### 5.5 Limitations of the current evidence

Many studies included a mix of surgical and non-surgical patients, and their findings may not be fully applicable to MSIs. It is noteworthy that dedicated research on orthopaedic patients remains limited; nevertheless, the existing evidence may be reasonably extrapolated to this vulnerable population.

While this scoping review provides valuable insights and a broad overview of the existing literature related to isolation measures in orthopaedic practice, there are several important limitations to note. Firstly, most studies were observational, introducing bias and limiting causal inference. Secondly, most studies also lacked a clear comparison group, making it difficult to draw definitive conclusions about the direct impact of isolation measures. Furthermore, isolation measures were often implemented alongside other infection control strategies, such as enhanced hand hygiene, and screening protocols, making it difficult to isolate the specific effect of isolation itself. Moreover, the diversity in isolation protocols, including the implementation of single-room isolation versus cohort isolation, made it challenging to determine the most effective strategy. Thirdly, most of the included studies focused on MRSA, while other pathogens were less frequently examined, which may limit the generalizability of the findings to a wider range of infections.

Given the escalating threat of antimicrobial resistance, it is essential that our recommendations be continually re-evaluated and updated to reflect the evolving evidence and ensure optimal patient care (GBD 2021 Antimicrobial Resistance Collaborators, 2024).

## 6 Conclusions

There is insufficient evidence to support the routine use of dedicated septic wards in orthopaedic practice, a strategy that was abandoned in many countries following the introduction of standard precautions in 1996 (Garner, 1996). Additionally, evidence relating to the routine isolation of patients with MSIs, especially those caused by non-MDROs, is limited even when these patients share a room with individuals undergoing elective orthopaedic procedures. Effective infection control can be achieved through hospital-wide strategies, provided appropriate preventive measures are in place. Isolation practices should be selectively applied and tailored to local resistance profiles and transmissibility of specific pathogens to balance infection prevention with optimal resource utilization. Ensuring effective care and infection control for MSI patients may be better achieved through management in specialized centres rather than by establishing separate surgical septic wards.

## Data Availability

The data used to support the findings of this study are included in the article (Table 2 and 3).
